# Miniature Mesa Extension for a Planar Submicron AlGaN/GaN HEMT Gate Formation

**DOI:** 10.3390/mi13112007

**Published:** 2022-11-18

**Authors:** Moath Alathbah, Khaled Elgaid

**Affiliations:** 1 School of Engineering, Cardiff University, Cardiff CF24 3AA, UK; 2 College of Engineering, King Saud University, Riyadh 11451, Saudi Arabia

**Keywords:** AlGaN/GaN HEMTs, device isolation, AlGaN/AlN/GaN HEMTs, planar gatefeed, gate leakage, HEMT mesa etch

## Abstract

In this letter, a novel approach is presented to overcome issues in AlGaN/GaN high electron mobility transistors (HEMTs), such as metal discontinuity of the gate stemmed from conventional mesa isolation. This usually requires a careful mesa etch process to procure an anisotropic mesa-wall profile. An alternative technique is the use of ion implantation for device isolation instead of conventional mesa for a planar device formation. However, ion implantation is a costly process and not always easily accessible. In this work, the proposed method is to simply extend the mesa below the gate just enough to accommodate the gatefeed, thereby ensuring the entire gate is planar in structure up to the gatefeed. The newly developed device exhibited no compromise to the DC (direct current) and RF (radio frequency) performance. Conversely, it produced a planar gate configuration with an enhanced DC transconductance (approximately 20% increase is observed) and a lower gate leakage while the etch process is considerably simplified. Similarly, the RF transconductance of proposed device (device B) increased by 80% leading to considerable improvements in RF performance.

## 1. Introduction

**Figure 1:**
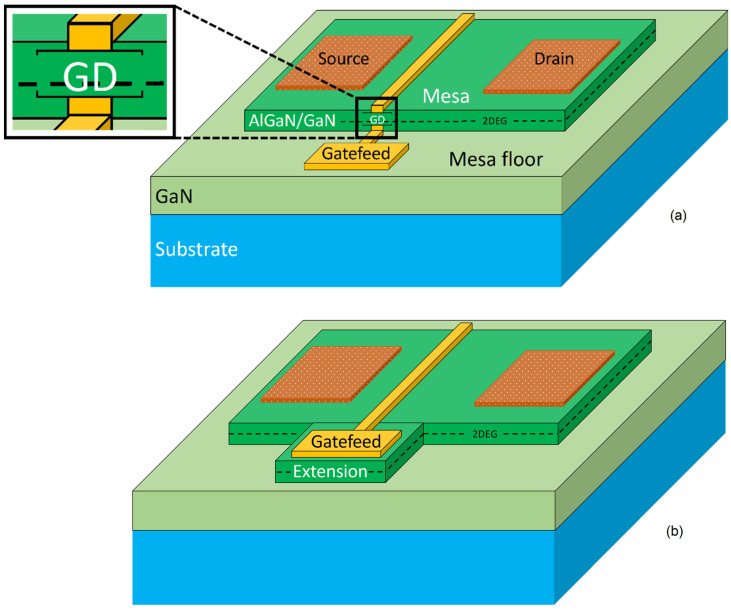
Conventional gallium nitride (GaN)-based high electron mobility transistor (HEMT) device with its gatefeed on mesa floor (fin-like gate) with the common issue of gate discontinuity (GD) shown at the mesa edge (**a**), and the proposed design of mesa extension for gatefeed (**b**).

Gallium nitride (GaN) is one of the most attractive semiconductors due to its inherent characteristics, such as electron mobility and saturation velocity, enabling the feasibility of high-power devices at microwave frequency with a considerably enhanced power density [1,2,3,4,5,6]. Currently, GaN-based HEMT devices fabrication includes a device isolation step performed by a conventional mesa etch or ion implantation to suppress the two-dimensional-electron-gas (2DEG) and electrically insulate devices. Although a three-dimensional (3D)-structure device is obtained due to physical removal of material, the former (mesa) is widely used for cost purposes. However, the introduction of a nonplanar structure in HEMTs results in a high gate leakage current, which degrades noise performance in RF amplifiers and can drive the device into breakdown prematurely [7,8]. This is because of the presence of a mesa sidewall in the conventional mesa-isolation process, the metal gate and/or the passivation layer come into contact with the 2DEG of the AlGaN/GaN HEMT structure, resulting in higher gate leakage current and non-uniformity in buffer breakdown voltage with passivation layer [9,10]. Additionally, there is possible metallic discontinuity of the gate at the mesa edge, especially at a small gate length *L_g_* ≤ 1.0 µm as shown in Figure 1a. Besides, in a mesa isolation, etch depth is critical for reproducible, optimized device and circuit performance. Under-etching causes current leakage and poor pinch-off of HEMTs, affects bias levels in circuits, and alters transmission line impedances [11]. In contrast, ion implantation isolation preserves the planar structure of the device but at a considerably higher cost [12]. Additionally, near-surface ion implants greatly degraded the resistivity of the damaged GaN. The considerable decrease in resistivity of the implant-damaged GaN after annealing at relatively low temperatures would most likely limit the use of ion implantation for lower temperature applications [13,14]. Other researchers have examined alternative methods, such as thermal oxidization, as reported in [15,16]. However, this process can affect the 2DEG beneath the actual device due to high temperature sample exposure for long durations, up to 900 °C and 30 min, respectively. Finally, as a technique to mitigate the metallic gate-discontinuity and leakage complications, air bridging the gate across to the bond pads has also been investigated as reported in [17,18,19]. Nevertheless, the process is complex [12] and can reduce device reproducibility and yield.

In this paper, a novel approach is provided to overcome the existing tradeoff between cost and 3D-structure disadvantages. A miniature extension from the mesa is employed to accommodate the gatefeed, which is the interconnect between the gate itself and the measurement bond pads (or transmission lines in integrated circuits). This, as a result, ensures a gate continuity, integrity, and reduces the gate leakage current since the critical section of the gate metal is positioned planarly on the mesa/extension in its entirety as illustrated in Figure 1b. Therefore, a direct contact between the gate and the 2DEG is avoided. Moreover, the bond pads or transmission lines are large features with a Si_3_N_4_ passivation underneath, hence a metal discontinuity and a direct 2DEG contact, respectively, are implausible to be present. The fabricated devices have the following dimensions: a device width of 125 µm, a gate foot length of 1.0 µm, a gate head of 1.5 µm, a total source-drain separation of 5 µm, a 1.5 µm of gate-source spacing and a 2.5 µm gate-drain distance. All the devices in this work have two gate fingers.

## 2. Materials and Methods

### 2.1. Material Growth

The devices in this work were fabricated using AlGaN/AlN/GaN epitaxy grown on 6-inch p-type low resistivity silicon (LR-Si) substrate by metal organic chemical vapor deposition (MOCVD). The GaN epilayer consists of a 200 nm AlN nucleation and a 750 nm graded buffer of AlGaN, both of which are applied to accommodate the lattice mismatch between the LR-Si substrate and GaN epitaxy to reduce the bow condition on the surface. Subsequently, a 1400 nm Fe-doped GaN graded buffer and channel, a 1 nm AlN spacer, a 25 nm Al_x_Ga_1-x_N barrier with Al composition (x) of 25% and finally capped with a 2 nm GaN to further manage the tensile strain on the barrier. The wafer from end-to-end is crack free with a maximum concave bow of only 20 µm, which was acquired after exposure to a high temperature around 1050 °C, during growth, followed by a cooling process.

### 2.2. Device Process

**Figure 2:**
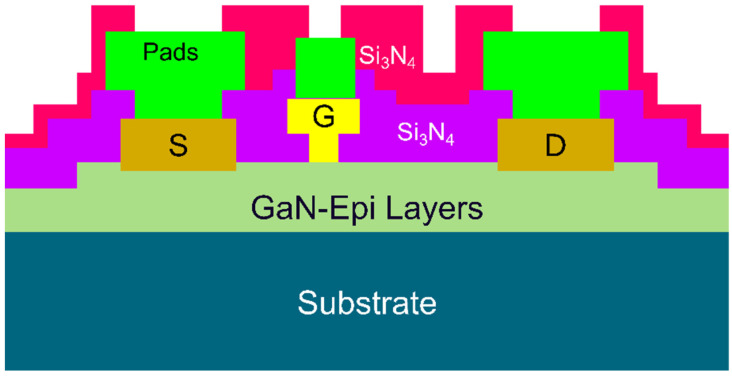
GaN HEMTs cross-section view for the fabricated devices.

In this work, all levels of device definition were realized using photolithography. The process commences with the fabrication of alignment markers. After that, the ohmic contacts (source/drain) were realized using the standard AlGaN/GaN HEMT metal scheme (Ti/Al/Ni/Au) and annealed at 790 °C for 30 s in N_2_ atmosphere. Next, mesa isolation at a depth of 200 nm was applied to remove the active layers between devices using a chlorine-based mixture of gases in an Inductively Coupled Plasma-Reactive Ion Etching (ICP-RIE) tool. After that, a 100 nm S_3_iN_4_ a blanket passivation was deposited by method of plasma enhanced chemical vapor deposition (PECVD) at 300 °C. The purpose of the first passivation layer is to suppress surface leakage and trappings, which can lead to a device unreliability. Then, gate footprints were realized using a low-damage SF_6_/N_2_ plasma etch to remove the Si_3_N_4_ passivation from underneath the gate foot. The metal (Ni/Au 20/200 nm) of the gate and its feed were then evaporated to form the Schottky contact of the device. Finally, after a second S_3_iN_4_ blanket passivation and openings etch, the bond pad metal was deposited for measurement using Ti/Au metal-stack with a metal thickness of 50/700 nm. The second passivation layer is applied only to protect the devices from oxidation and corrosion, which can help to extend the lifetime of the devices. The cross-section of the fabricated devices is given in Figure 2.

### 2.3. Proposed Devices Design

**Figure 3:**
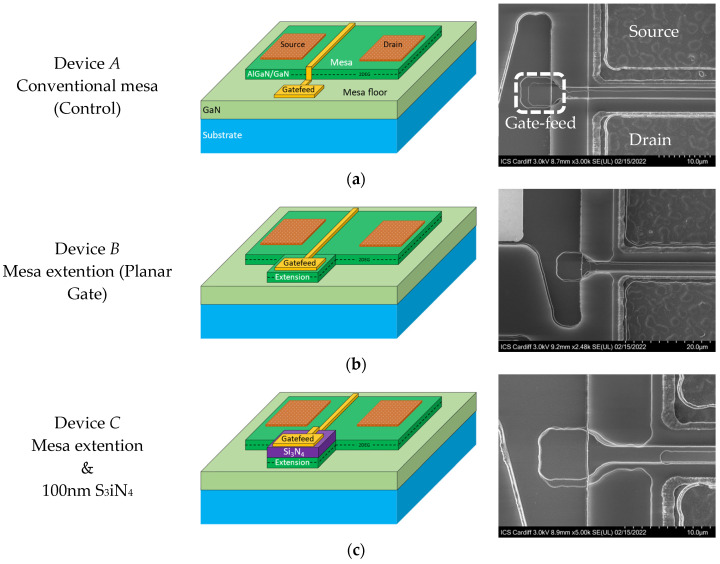
Three fabricated devices 3D visualization and scanning electron microscope (SEM) images, conventional (**a**), planar (**b**) and elevated by Si_3_N_4_ (**c**).

In this work, three device structures are fabricated as shown in Figure 3. The first one (device A) is the conventional HEMT, which has its gate-feed directly on the GaN semiconductor after etching the Si_3_N_4_ passivation. Device A is the control device to which the proposed devices results are compared to. The proposed device is fabricated into two different structures with respect to the location of the gate-feed. The first and the primary proposed device has its gate-feed directly on the proposed miniature mesa extension and the second one the gate-feed is elevated above the extension by a 100 nm of Si_3_N_4_, annotated by device B and C, respectively. It is worth noting that the footprint of the gate-feed of device B is situated directly on an active layer (i.e., the mesa extension) and it is wide in size (3.5 µm wider than the gate footprint). This ultimately results in a lower resistance path at the gate-feed of device B. Therefore, the fabrication of device C is necessary to suppress the lower resistance access point, obtained in device B, by adding an insulation layer below the gate-feed. Although this results in a non-planar device, nevertheless the gate has no direct contact with the 2DEG channel. This will enable for a clearer observation and characterization of the active layer influence on the performance of device B.

The mesa extension, as shown in Figure 3, is designed to accommodate the gate-feed. The length of the mesa extension is 13 µm from the actual device-mesa and the width is designed to be larger than the gate and the gate-feed by 0.5 µm on all sides to avoid any potential misalignment may be arising from the photolithography. Both proposed structures are compatible with the conventional GaN HEMTs process, therefore realizable without any additional fabrication requirements.

## 3. Results and Discussion

### 3.1. DC and RF Performance

On-wafer DC and RF measurements were performed using an Agilent semiconductor parameter analyzer (B1500A, Agilent Technologies, Santa Clara, CA, USA) and a microwave network analyzer (N5227A, Keysight Technologies, Santa Rosa, CA, USA), respectively. The latter was calibrated from 100 MHz up to 50 GHz with an off-wafer impedance standard substrate (ISS) calibration kit for a 100 µm pitch RF-probe by utilizing a Short-Open-Load-Through (SOLT) calibration procedure at a small signal RF excitation (−20 dBm).

**Figure 4:**
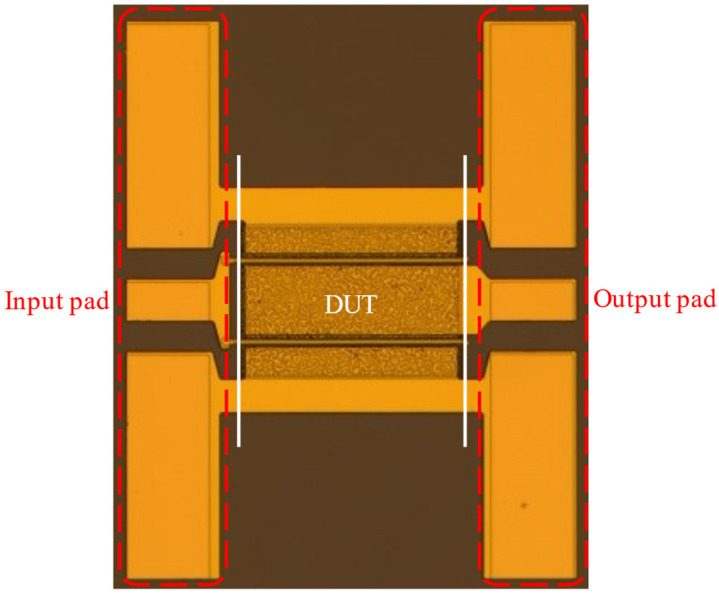
Device under test (DUT) and the input and output coplanar waveguide (CPW) pads.

To accurately assess the fabricated device at high frequency, a de-embedding process is executed to remove the pads parasitic impedances from the device under test (DUT) as shown in Figure 4. The de-embedding method begins with three measurements, the embedded-DUT (eDUT), an open and short “dummy” structures of the pads without the device. The de-embedding technique is carried out directly on the measurement system using a customized routine in Keysight PathWave Device Modeling software (IC-CAP 2020, Keysight Technologies, Santa Rosa, CA, USA), in which an algorithm is coded to mathematically remove the measurement bond-pads effects (the open and short) from the measured data.

**Figure 5:**
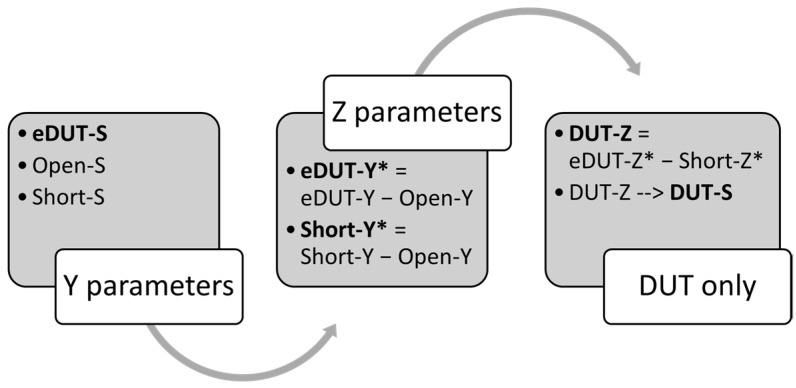
RF (radio frequency) measurements CPW pads de-embedding process flow, “S” in the first group refers to S-parameters.

All RF measurements are initially collected in a two-port S-parameters format. This is followed by conversion to Y-parameters for the three measurements. The Y-parameters of the open fixture is then subtracted from the eDUT and the short fixture measurements. Finally, the partial de-embedded data of eDUT-Y* and de-embedded short fixture (Short-Y*) are converted to Z-parameters, forming eDUT-Z* and Short-Z* respectively. The latter is then subtracted from the former resulting in a fully de-embedded data which is then converted back to S-parameters format (DUT-Z → DUT-S) for further mathematical manipulation. Figure 5 summarizes the process flow and the algorithm for the device de-embedding methodology utilized in this work.

**Figure 6:**
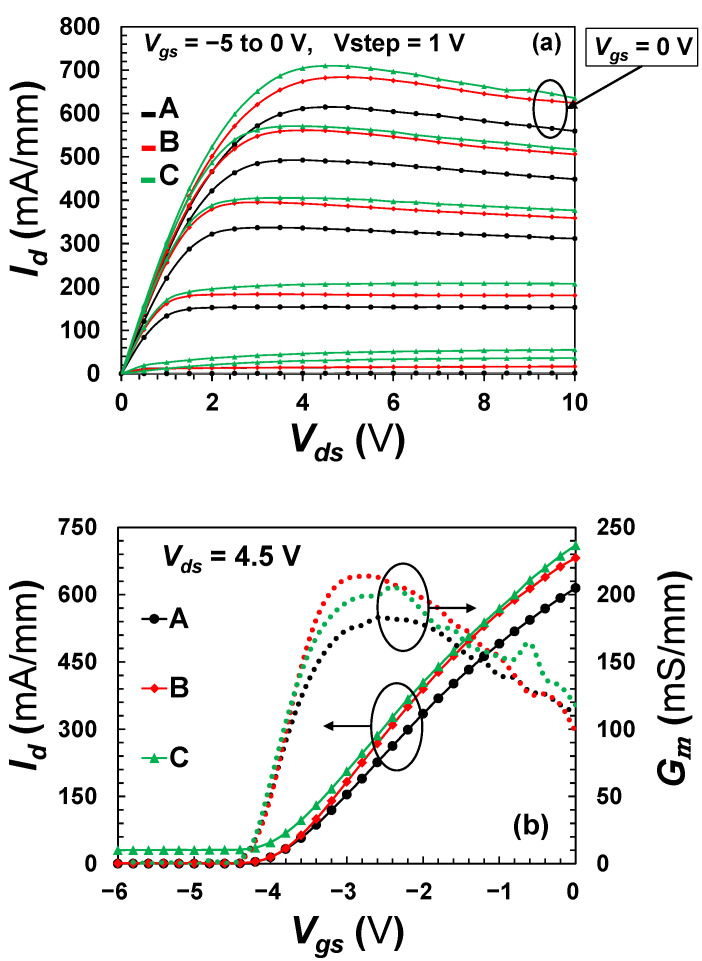
(**a**) I-V family curves of 2-finger 125 µm wide device, (**b**) the corresponding transfer characteristics of the three devices.

An assessment of *IV* characteristics on all three devices was performed to validate the influence of mesa extension on device performance in terms of output current, as shown in Figure 6a. The maximum drain current, *I_DSS_*, of the three devices is 692, 772 and 813 mA/mm for device A, B and C, respectively, obtained at *V_ds_* = 4.5 V. The increase of maximum current in the proposed devices is stemming from the reduction in the On-resistance (*R_ON_*) from 3.57 Ω⋅mm for device A to 3.23 and 3.05 Ω⋅mm for device B and C respectively. Further, a well pinch-off behavior is found in all devices at −4.2 V as depicted in Figure 6b. However, in Figure 6b also, a drain leakage is observed in device C evidenced by the drain current not approaching the zero point of the y-axis as the other devices. This drain leakage was observed in all measured C-type devices across the entire wafer. This is usually ascribed to traps in AlGaN/GaN HEMTs associated with electron injection from the gate and trapping either inside the AlGaN layer or at the surface close to the gate [20]. Nonetheless, since device A and B, which were fabricated on the same wafer, did not exhibit such issue with drain leakage, the aforementioned traps in the materials are unlikely to be the cause of the drain leakage in device C. Therefore, given that device C is the only device configuration with an insulation layer (100 nm of Si_3_N_4_) below its gate-feed, this can be considered as the leading trigger for the device drain current leakage (further analysis is given next in Section 3.2).

Additionally, at *V_ds_* = 4.5 V and *V_gs_* = −3 V, a maximum DC transconductance (*G_m_*) was obtained for the three devices. However, the proposed devices exhibited an increase of *G_m_* by almost 20% from 177 to 200 mS/mm, which can be attributed to the increase of the gate width (*W_g_*) by a total of 26 µm (2-finger × 13 µm ≈ 20% total width increase) due to the mesa extension as predicted from the following formula [21,22]:

(1) $$G_{m}=\frac{\varepsilon_{barrier}v_{sat}W_{g}}{\left(d_{barrier}+\Delta d\right)}$$

where *ε_barrier_* is barrier layer dielectric constant, *v_sat_* is the saturation velocity, *d_barrier_* is the barrier layer thickness, and Δ*d* is the effective distance of the 2DEG from the heterointerface.

**Figure 7:**
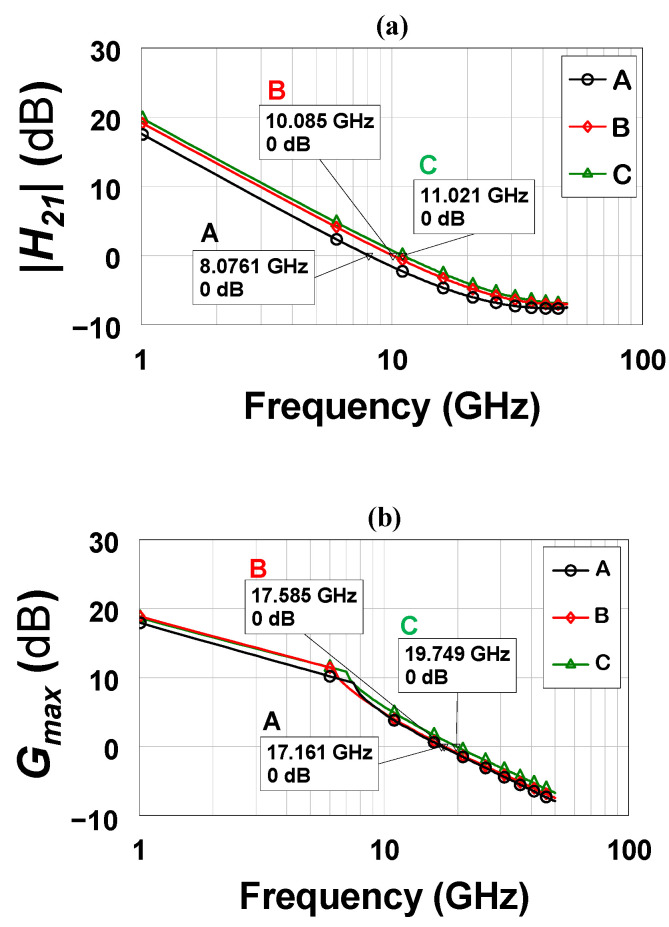
The small-signal gain characteristics current gain (**a**) and maximum available gain (**b**) obtained at *V_ds_* = 4.5 V and *V_gs_* = −2.5 V.

A comparison of RF performance was conducted to substantiate the impact of mesa extension on device performance in terms of device cutoff (*ƒ_T_*) and maximum oscillation (*ƒ_max_*) frequencies, which can be evaluated using Equations (2) and (3), respectively. Figure 7 shows a rise in *ƒ_T_* and *ƒ_max_* as the gatefeed location is altered triggered by the increase in RF transconductance (*g_m_*) and/or the decrease in gate-drain/source capacitances [23] extracted from a small signal model.

(2) $$f_{T}=\frac{g_{m}}{2\pi \left(C_{gs}+C_{gd}\right)}$$

(3) fmax=fT2[Rg+RinRds]12

where *C_gs_* is the gate-source capacitance, *C_gd_* is the gate-drain capacitance, *R_g_* is the gate resistance, *R_in_* is the input resistance and *R_ds_* is the output resistance.

Device B demonstrated the highest *g_m_* (108 mS) with an increase of 80% in comparison with the control device (device A) with only 60 mS of *g_m_*. Device C, on the other hand, showed a 25% increase in *g_m_*. Even though the proposed device exhibited the highest *g_m_*, a clear DC-to-RF dispersion is present manifested by the variations between DC and RF transconductances, i.e., *G_m_* and *g_m_*, respectively. This was the case for all of the fabricated devise across the entire wafer. This could be attributed to the quality of the passivation utilized in this work. GaN devices, in general, have been demonstrated to be extremely sensitive to various surface passivation and preparation procedures, indicating that trapped surface charge plays a substantial role in the dispersion [24,25].

In a field effect transistor (FET), the source of fringing capacitance between the electrodes are due to the electric field’s lines occurring between them. This electric field is present in the air (at the surface) and more strongly within the semiconductor, assuming there is no surface passivation, due to a higher dielectric constant (*ε_r_* ≈ 8.9 for GaN material). However, stronger electric field at the surface can occur if a passivation layer is deposited on the device leading to an increase of the overall fringing capacitance. For example, a 20% increase in fringing capacitance is obtained using a 30 nm of Al_2_O_3_ passivation as reported in [26]. In addition, knowing that the fringing capacitance (*C_f_*) is inversely proportional to the distance (*d*) between the electrodes as given in Equation (4).

(4) $$C_{f}=\frac{\varepsilon_{o}\varepsilon_{r}A}{d}$$

where *A* is the area of plates (the metallic surface of electrodes), *ε_o_* is the permittivity of free space and *ε_r_* is the relative permittivity of the material. Although, the inclusion of the mesa extension increases the distance between the gate-feed and the source/drain contacts from the GaN semiconductor side (by 200 nm), the distance at the surface is in fact reduced since they are on the same level plane with a 100 nm Si_3_N_4_ dielectric between them. This explains the increase in fringing capacitance acquired in device B. Nevertheless, device B exhibited a slight improvement in RF performance, in comparison with the control device, merely due to the improvement in *g_m_*. Device C, on the hand, showed the optimum enhancement in RF performance with 35% and 15% increase in *ƒ_T_* and *ƒ_max_*, respectively. This is primarily the result of the reduction in fringing capacitance due to the presence of Si_3_N_4_ below the gate-feed. This further increases the distance between the gate-feed and the source/drain electrodes resulting in a lower electric coupling and thereby a lower capacitance at the semiconductor and at the surface simultaneously. A detailed comparative study between mesa and ion-implantation isolation with respect to RF performance is reported in [27]. The study shows a reduction in the gate fringing capacitance by 5.6% and an increase in *g_m_* by more than 11% for ion-implanted device in comparison to a mesa etched one. These attainments resulted in an average increase of 17% in *ƒ_T_*. The authors attributed the improvement obtained by ion-implantation isolation in part to defects generated at the ICP-etched mesa edge and/or even on the mesa floor surface due to ion bombardment during the dry etch process [27,28].

Summary of DC and RF parameters for the fabricated devices are included in Table 1. No compromise in performance is observed for the fabricated devices. This manifests that the proposed mesa extension is not presenting any deterioration to the parameters of the devices. In fact, the proposed devices can offer a higher power density in comparison with the conventional one. Almost 10–14% increase in power density is obtained by the proposed devices. This is equal in percentage to the amount of reduction achieved in *R_on_*.

### 3.2. Gate and Drain Leakage Currents

**Figure 8:**
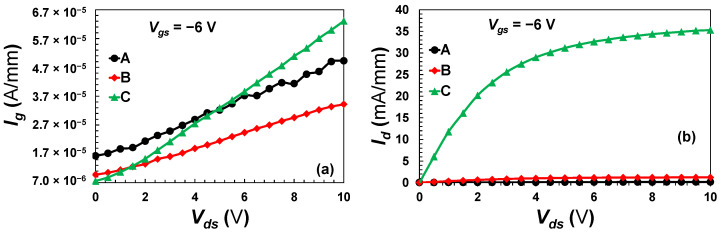
The Off-state gate leakage current (**a**) and the drain leakage current below pinch-off (**b**) as a function of the drain bias.

An evaluation of gate and drain currents on the devices was performed to validate the effect of mesa extension on device performance in terms of leakage, as demonstrated in Figure 8. The off-state gate leakage current of all devices is shown in Figure 8a with a drain voltage sweep from 0 to 10 V and a gate voltage of −6 V. Across the entire drain bias, device A exhibited an order of magnitude larger gate leakage currents than device B. The proposed planar gate device, with mesa extension, revealed lower gate leakage currents as expected compared to the conventional one, which is comparable to the decrease obtained by 131Xe^+^ ion-implantation as given in [29]. This is due to the gate in the conventional device being in a direct contact with the 2DEG channel [30,31] while the gate in the planar device has the AlGaN barrier acting as an insulator between the gate and the 2DEG. However, device C demonstrated a low gate leakage at lower drain voltages and vice versa at higher biasing with an order of magnitude of leakage between the minimum and maximum *V_ds_*. This is attributed to the device’s poor linearity [31] evidenced by the irregular shape of the *G_m_* displayed by device C as shown in Figure 6b. Additionally, due to the use of PECVD in the formation of Si_3_N_4_, a hot hole injection and trapping is present at the insulator/semiconductor interface [32]. Takatani et al. further explain that the hot holes produced by impact ionization at the gate’s drain end are injected and trapped at the insulator/semiconductor interface between the gate and the drain, thus increasing the electric field at the gate-edge. This is attributed to a couple major drawbacks of the PECVD method: (1) generated films contain large concentrations of bound hydrogens, and (2) surface damage can be induced by the bombardment of high-energy ions, resulting in a high density of interface states [33].

Subsequently, with the same bias conditions, the off-state drain leakage current of the fabricated devices is also presented in Figure 8b. Device A, B and C exhibited 0.19, 1.24 and 35.3 mA/mm of drain leakage, respectively, at a drain voltage of 10 V. This is primarily due to the degraded gate controllability over the drain-source channel as the distance between the gate edges and the 2DEG is enhanced [34,35].

**Figure 9:**
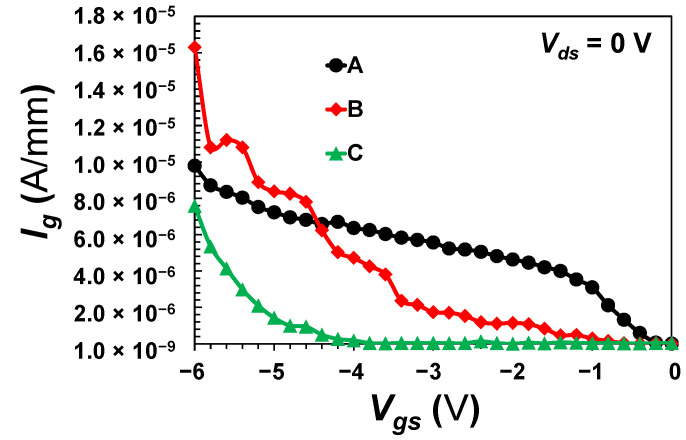
The gate leakage as a function of the applied voltage at the gate terminal.

Finally, the devices were swept at the gate terminal from −6 to 0 V without drain bias, i.e., *V_ds_* = 0 V. Both proposed devices (B and C) exhibited the minimum gate leakage particularly between −4.5 and −0.5 V of a gate bias, whereas the control device demonstrated an order of magnitude higher gate leakage at the same bias level as depicted in Figure 9. This is mainly due to the direct contact between the gate electrode and the 2DEG as in device A. Moreover, due to the absence of a drain bias, device C showed an optimum suppression of gate leakage in comparison with the other devices. However, the leakage is considerably enhanced in device C when a drain bias is applied caused by the hot hole injection and the trapping as aforementioned.

## 4. Conclusions

In this work, a newly developed structure is proposed to overcome mesa etch drawbacks, such as mesa sidewall profile, common gate discontinuity, and gate leakage originated from a gate direct contact with 2DEG. The proposed structure requires a miniature extension of the mesa to deposit the gatefeed, thereby ensuring a fully planar gate formation. This, as a result, reduces the etching complexity and circumvents the direct contact between the gate and the 2DEG. With more than an order of magnitude of gate leakage suppression, the proposed device also showed a better DC and RF performance than the conventional device. Further investigation, however, is necessary to fully understand the rise of the drain off-state current of the planar device and to find a method to enhance the gate controllability of the device.

## Figures and Tables

**Table 1:** DC (direct current) and RF (radio frequency) parameters for the fabricated three devices with 1 µm gate length and 2-finger of 125 µm width achieved at *V_ds_* = 4.5 V and *V_gs_* = −2.5 V.

Parameter	Device (A)	Device (B)	Device (C)
*I_DSS_* (mA/mm)	692	772	813
*P_density_* (W/mm)	6.5	7.1	7.4
*V_Pinch-off_* (V)	−4.0	−4.0	−4.0
*R_ON_* (Ω⋅mm)	3.57	3.23	3.05
*G_m_* (mS/mm)	177	211	198
*ƒ_T_* (GHz)	8.08	10.08	11.02
*ƒ_max_* (GHz)	17.16	17.58	19.75

## Data Availability

The data presented within this paper is available on a reasonable request from the first author.
